# P23 - Boy with severe atopic dermatitis treated with omalizumab

**DOI:** 10.1186/2045-7022-4-S1-P78

**Published:** 2014-02-28

**Authors:** Evangelia Stefanaki, Emmanouel Manousakis, Vassiliki Aggelakou, Amalia Tsilimigaki

**Affiliations:** 1 Pediatric Alllergy Outpatients Clinic, Venizelion General Hospital, Heraklion, Greece; 2 Allergy Department, 2nd Pediatric Clinic, University of Athens, Children’s Hospital “P. and A. Kyriakou”, Athens, Greece

## Introduction

Atopic dermatitis (AD) is a common inflammatory, itching skin disease. In a subgroup of patients, the disease is recalcitrant to topical therapy and systemic treatments become necessary. Biological agents are a new approach in AD therapy.

## Methods

We describe the course of a case of severe AD under treatment with omalizumab.

## Case report

Our patient is a boy 9.5 years old who has AD, vernal conjunctivitis, persistent rhinitis and asthma and is sensitized mainly to dust mites.

His AD exists since infancy but became more intense since 3 years old. In 2010 he used intense moisturizing treatment and high-potency topical corticosteroids both pro actively and on demand and under these conditions his SCORAD levels were 21-32%. In the beginning of 2011 he had a persistent deterioration (SCORAD 46%) despite intense treatment. He even required admission. On May 2011 oint tacrolimus 0.1% was also started on a daily basis with only a minor improvement (SCORAD 32%). Parents were hesitant to start systemic therapy with cyclosporine and under these circumstances consultation was given from Peadiatric Allergy Unit in Athens Paediatric Hospital A. and P. Kyriakou to start with omalizumab 750mg subcutaneously monthly. He started treatment in July 2011 and since then his condition improved dramatically. His SCORAD levels fell under 10% and gradually he also limited the use of corticosteroids and tacrolimus. Last season he stopped totally tacrolimus and stayed stable.

His asthma is mainly viral induced and although under treatment with omalizumab and inhaled fluticasone he still had 3 mild episode between Sep 2011-May 2012. He stopped fluticasone in July 2012 and since then he has no episode. He is also free of symptoms between episodes with normal spirometry.

His rhinitis remains mild persistent despite treatment.

His vernal conjunctivitis started in 2010, was severe persistent but it is under control since 2012.

## Conclusions

Our case report suggests that omalizumab might have a place in the treatment of AD refractory to the standard therapy. Our result should be confirmed in controlled studies performed in large samples.

